# Agricultural land use and ensuing eutrophication both shape parasitic trematode communities in rural African lakes

**DOI:** 10.1098/rspb.2025.0070

**Published:** 2025-06-04

**Authors:** Cyril Hammoud, Bert Van Bocxlaer, Dirk Verschuren, Julius Tumusiime, Christian Albrecht, Wannes De Crop, Casim Umba Tolo, Tine Huyse

**Affiliations:** ^1^ Limnology Unit, Department of Biology, Ghent University, Ghent, Flanders 9000, Belgium; ^2^ Department of Biology, Royal Museum for Central Africa, Tervuren, Flanders 3080, Belgium; ^3^ Coastal Systems, Royal Netherlands Institute for Sea Research, Den Burg 1790 AB, The Netherlands; ^4^ CNRS, University of Lille, UMR 8198 Evo-Eco-Paleo, Lille, Hauts-de-France 59000, France; ^5^ Department of Biology, Mbarara University of Science and Technology, Mbarara, Western Region 1410, Uganda; ^6^ Department of Animal Ecology and Systematics, Justus-Liebig-Universitat Giessen, Giessen 35392, Germany; ^7^ Laboratory of Biodiversity and Evolutionary Genomics, KU Leuven, Leuven, Flanders 3000, Belgium

**Keywords:** biodiversity, agriculture, One Health, parasites, Uganda

## Abstract

Land use is a major driver of biodiversity loss, but how it impacts parasite communities is scarcely documented. Crater lakes and their catchments in rural western Uganda greatly vary in their intensity of anthropogenic disturbance, thus providing an opportunity to assess the effects of land use on snail-borne parasitic trematodes. We applied state-of-the-art molecular biomonitoring to 2385 *Bulinus tropicus* snails from 34 lakes to detect and genotype trematode infections. The 45 trematode taxa recovered infect a wide range of final vertebrate hosts, and some can cause health burdens of significant public importance. Using constrained ordinations and generalized additive models, we found that *B. tropicus* reaches peak abundance in lakes with catchments partly under agriculture, whereas trematode infections increase with *B. tropicus* abundance and peak at intermediate aquatic productivity. Trematode diversity also increases with aquatic productivity, levelling off only in the most productive lakes. These relationships likely reflect the higher abundance and variety of final hosts sustained by more productive lakes. Finally, we found that land use affects trematode community composition, with more livestock parasites and less bird parasites occurring in agricultural catchments. Our results indicate that both land use and lake eutrophication affect the distribution of hotspots for parasitic disease transmission.

## 1. Introduction

Human-mediated environmental changes are causing a global biodiversity crisis, threatening ecosystem functioning and human well-being [1]. Understanding exactly how those changes affect biodiversity is hence an important focus of current ecological research. Widespread loss of natural habitats and alteration of hydrological and nutrient cycles are among the most influential drivers of biodiversity loss [2], and their effects differ greatly among different groups of organisms [3]. However, our understanding of how and to what extent anthropogenic disturbances impact organisms with a parasitic lifestyle is highly fragmentary. To date, studies have predominantly focused on a few medically or economically relevant parasite species and on how decreases in the abundance and diversity of final hosts affect parasite transmission [4,5]. Yet besides causing important diseases to humans and livestock, metazoan parasites represent approximately 40% of animal biodiversity on Earth [6], and they provide key ecosystem functions such as regulating host abundance and mediating competitive interactions within food webs [7,8]. Consequently, alteration of parasite populations by anthropogenic activity are likely to affect both the host species and other interacting taxa, increasing the risk of ecosystem destabilization. Apart from a few pertinent studies [9,10], the compound effects of multi-faceted anthropogenic ecosystem disturbance on parasite diversity and especially parasite community composition remain largely undocumented. While some authors [8,11] advocate prioritizing a strategic agenda for parasite conservation, any conservation measure will be handicapped by lack of data on exactly how human activities affect parasite diversity and community composition at the ecosystem level. To the extent that this knowledge gap can be attributed partly to the challenge of gathering data on parasite communities in a comprehensive manner, new methods for genetic screening are now easing this bottleneck on progress. In this study, we use a powerful and scalable workflow for high-throughput genetic sequencing [12] to analyse the compound impact of human-mediated environmental change on communities of parasitic trematodes inhabiting small tropical lakes in rural western Uganda.

Trematodes are parasitic flatworms with complex life cycles that generally use an aquatic snail as the first intermediate host and a variety of invertebrates and vertebrates (fishes, reptiles, birds and mammals, including humans and livestock) as secondary and final hosts [13]. Therefore, depending on their stage of development, many trematode species inhabit both freshwater lakes and adjacent terrestrial ecosystems. Our genetic screening method [12] enables detailed taxonomic and structural characterization of a lake’s trematode community by simultaneously genotyping all parasites infecting individuals of the host snail, so that sampling snail populations from multiple lakes allows documention of the occurrence and distribution of individual trematode species across an entire study region. However, to reveal the precise effects of human activity on trematode diversity and community composition in real ecosystems, a study system is required where confounding factors have limited influence. Such conditions are provided by approximately 65 small, tropical freshwater lakes in rural western Uganda, which each occupy a volcanic ‘maar’ crater basin and are thus surrounded by a topographically confined catchment [14]. This facilitates quantifying the intensity of local human activity as proportional to the fraction of catchment area where natural vegetation (mostly lowland tropical forest) has been replaced by agriculture (mostly crop fields). Importantly, variation among these lakes in the natural environmental factors controlling aquatic productivity and habitat diversity (such as climate regime, hydrology and water chemistry) is modest, so that variation in aquatic productivity (i.e. trophic status) is also mainly controlled by differences in the intensity of agricultural land use across catchments [14,15]. Located in the Albertine Rift Valley, these lakes and their catchments constitute critical natural elements within a prominent African hotspot of bird and mammal diversity [16], which like tropical biodiversity as a whole [17] has become particularly vulnerable to the spreading and intensification of land use associated with increasing demographic pressure (from approx. 80 people km^–^² in 1990 to approx. 250 people km^–^² in 2020). Western Uganda being a rural region where the principal livelihood strategies are mixed subsistence farming and cattle herding, this has expectedly also increased the health burden owing to snail-borne diseases such as schistosomiasis and liver fluke disease [18].

Pursuing a One Health approach [19], this study assessed how land use influences communities of parasitic trematodes in the crater lakes of western Uganda, both directly and by influencing the distribution and abundance of their intermediate host *Bulinus tropicus*, a pulmonate (air-breathing) aquatic snail of the Bulinidae family. We analysed the abundance, taxonomic diversity and composition of the trematode communities infecting *B. tropicus* snails in relation to variation among lakes in aquatic habitat conditions and in the fraction of the adjacent terrestrial ecosystem impacted by agricultural activity. In this region, *B. tropicus* is the most common intermediate host [20] and is known to transmit trematodes to both wildlife and livestock [21]. The genetic diversity of *B. tropicus* in the crater lakes of western Uganda is high overall, likely as a result of multiple colonization events from various regional sources [20]. Although the different genetic makeup of *B. tropicus* populations among lakes may potentially cause slight variation in their susceptibility to trematode infections, this variation is unlikely to align with the gradient of anthropogenic impact intensity across all lakes and is therefore not expected to be a confounding factor in this study. Based on earlier work [22,23], we expected that the enhanced aquatic productivity (eutrophication) caused by excess nutrient input from land under cultivation would increase host snail abundance and thus promote higher trematode abundances. We hypothesized that trematode diversity and community composition might be negatively affected, however, reflecting a predominantly adverse effect of intensifying local human activity on the trematodes’ access to their final hosts.

## 2. Material and methods

### (a) Environmental characterization

**Figure 1.**
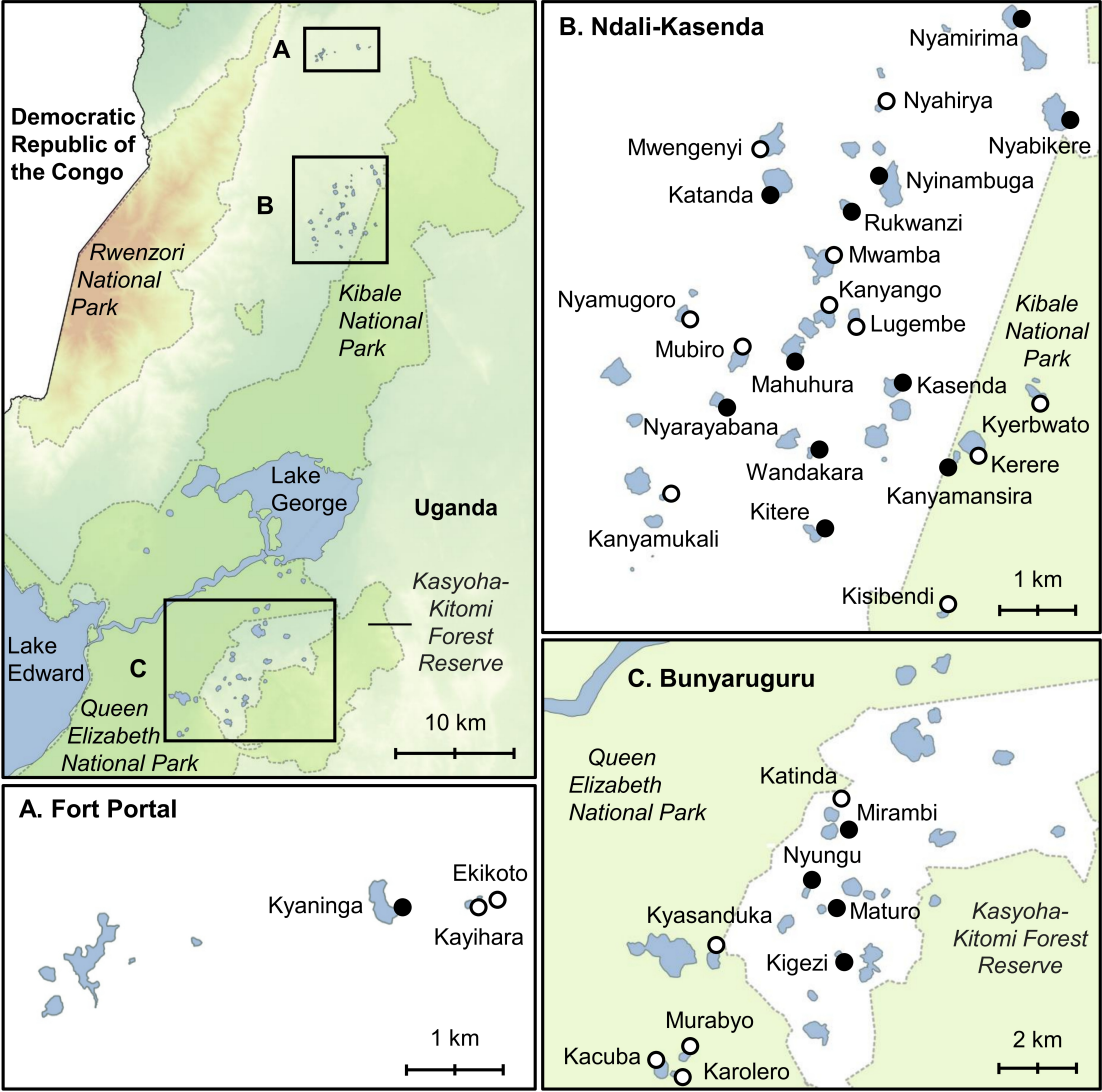
The Edward–George extension of the Albertine Rift Valley in western Uganda with topographic setting and maps of the three surveyed crater-lake clusters. Protected areas (national parks and forests) are delimited by light grey stippled lines and shaded in darker green. Only the 34 studied lakes are labelled by name. Filled circles mark the 16 lakes where ≥50 *B. tropicus* specimens were collected, allowing genetic characterization of the local trematode community.

The Albertine Rift Valley in western Uganda harbours *ca* 80 small lakes that were formed when volcanic eruptions within the past *ca* 50 000 years [24] created steep-sided crater basins that subsequently filled with groundwater and local rainfall [25]. We surveyed 34 of the *ca* 65 freshwater lakes (conductivity 143−1171 µS cm^−1^ [26]), selected to maximize the gradient of land use intensity in their crater catchments, which ranged from fully covered by (semi-)natural vegetation to having been entirely converted for agriculture (electronic supplementary material, table S1). The studied lakes are distributed across the Fort Portal, Ndali-Kasenda and Bunyaruguru clusters (figure 1), where modest altitudinal (*ca* 500 m) and hydroclimate gradients translate into slight variation in natural vegetation from moist deciduous lowland forest with small patches of natural grassland (‘wet savannah’) within the rift valley to contiguous moist evergreen lowland forest on the rift shoulders. In each lake, we collected individuals of *B. tropicus* from a shoreline stretch of approximately 20 m that has good accessibility and therefore often constitutes an access point for the local population and livestock. At each site, surface-water conductivity and pH were recorded using a Hydrolab Quanta multimeter to confirm freshwater conditions and to avoid low-pH habitats that are naturally less suitable for aquatic snails. Additionally, we retrieved high-quality environmental data on each of the sampled lakes from the literature. Estimates of aquatic productivity (and associated lake trophic status) are based on multi-seasonal measurements of the ubiquitous photosynthetic pigment chlorophyll-a (Chl-a) as proxy of phytoplankton biomass in 18 lakes [15] and the documented relationship between water transparency and Chl-a concentration [14] in the other lakes (details in electronic supplementary material, table S1). Chl-a values were log_10_-transformed to reduce skewness [15]. Surface-water transparency data are provided by [26] or [27] for all lakes except Nyamirima, for which missing data were replaced with the median values of the entire dataset. The average slope of terrain within each crater basin [28] was used as proxy for the width of the littoral zone providing shallow-water habitat to aquatic snails and accessibility to terrestrial animals and livestock (i.e. the zone where parasite transmission occurs). This slope also controls susceptibility to soil erosion [28], which in turn affects aquatic ecosystem functioning and productivity [29]. Estimates of total sediment influx to the lakes resulting from soil erosion, as proxy for the compound impact of land use on the aquatic ecosystem (through excess nutrient input and siltation), were obtained from [28]. These sediment yields were calculated by applying the Revised Universal Soil Loss Equation [30] and a sediment delivery distributed model [31] to Sentinel-2A satellite images [32]. For lakes with inflowing streams, sediment yield estimates were averaged over the range reported by [28]. Specific data on the distribution of different types of natural and anthropogenic land cover in each catchment, as inferred from Sentinel-2A satellite images [28], were used to calculate the fraction (% area) of each catchment occupied by (semi-)natural vegetation (forest, woodland or grassland), timber plantations (eucalyptus and pine) and crop fields (banana, coffee, manioc, maize and vegetable gardens) as proxy for variation in land use intensity. Finally, we retrieved reanalysis data on mean annual precipitation (MAP) and mean air temperature (MAT) at each study site from WorldClim [33].

### (b) Sampling of intermediate host snails

In February 2019, we collected molluscs (snails and bivalves) from the 34 selected lakes by scooping nearshore aquatic vegetation and unconsolidated sediments for 45 min and down to *ca* 1 m water depth. All collected specimens were sorted, counted and identified to species or genus level using shell-diagnostic features [21]. They were then sacrificed by heat shock at *ca* 70°C for *ca* 45 s and preserved in 80% analytical-grade ethanol. We obtained in total 8865 molluscs from 32 of the surveyed lakes; none were found in Katinda and Kisibendi. For the purpose of this study, we focused on *B. tropicus*, the most abundant species (3262 specimens; 36.8%) and found at 24 lakes. To ensure that our findings are consistent with the known regional distribution of *B. tropicus*, we compared our field data with those of two earlier snail surveys [20,27]. Results were highly similar, except that we did not find *B. tropicus* in four lakes (Kanyamukali, Kayihara, Murabyo and Nyahirya) where it had been recorded before at very low abundance. For analyses of *B. tropicus* distribution in relation to environmental factors, we made our dataset representative of all three surveys by assuming that a nominal number of five *B. tropicus* specimens were found at these four lakes.

### (c) Genetic characterization of trematode communities

Genetic screening was limited to the 16 lakes where >50 *B*. *tropicus* specimens were collected to ensure accurate characterization of local trematode diversity and community composition. In lakes with abundant *B. tropicus*, we limited infection screening to *ca* 200 randomly selected specimens per lake, except at Kasenda where the trematode community had already been characterized earlier by screening of 419 *B. tropicus* specimens [34]. In total, we screened 2385 *B. tropicus* (73% of those collected) for trematode infection with a multiplex polymerase chain reaction [35]. Specifically, all soft tissues were separated from the shell, homogenized with a sterilized scalpel and subjected to genomic DNA extraction using the E.Z.N.A. Mollusc DNA Kit (OMEGA Biotek, Norcross, GA, USA). DNA extracts were diluted in ultrapure water at 1 : 10 concentration and screened for the presence of trematode DNA using multiplex PCR. Subsequently, we genotyped the trematodes of all infected *B. tropicus* (*n* = 861, or 36.1% of those screened) with high-throughput amplicon sequencing (HTAS; [12]). This HTAS workflow starts with a single multiplex PCR reaction in which five trematode marker genes are simultaneously amplified: respectively two, one and one fragments of the mitochondrial genes cytochrome c oxidase subunit 1 (cox1 I, cox1 II), NADH dehydrogenase subunit 1 (NAD1) and cytochrome b (cytb) and one fragment of the nuclear internal transcribed spacer 2 (ITS2). Genomic library preparations were performed as described in [12], during which amplicons were tagged with sample-specific identifiers, pooled and sequenced using Illumina MiSeq v3 technology. The sequencing data were subjected to quality control and processed into amplicon sequence variants (ASVs) using DADA2 [36]. Our quality-controlled ASVs are deposited in GenBank under accession numbers OQ548105–OQ549888 (ITS2); OQ543469–OQ543563 (cox1 I); OQ606413–OQ606758 (cox1 II); OQ573734–OQ574607 (NAD1) and OQ574626–OQ575328 (cytb). DNA vouchers are deposited at the Royal Museum for Central Africa (Tervuren, Belgium) under BE_RMCA_MOL_DNA codes numbered 000001 to 002472. Finally, we used these sequences to identify the trematode species present via BLAST [37] and phylogenetic analyses using the methodology detailed in electronic supplementary material, text S1.

### (d) Trematode community ecology

Individual *B. tropicus* snails may be infected by one or more trematode species, which together comprise an infracommunity [38]. We characterized trematode infracommunities from all ASVs recovered per infected snail. As trematodes reproduce asexually within their snail host, we interpreted recovery of multiple ASVs belonging to the same trematode species from one individual snail as cases of co-infection by different strains of that species. Using these infracommunities, we reconstructed the total trematode communities (i.e. component communities) infecting the *B. tropicus* population of each studied lake. The composition of these communities was tabulated in the form of lake-specific abundance matrices, which we used to estimate local trematode species richness and Hill–Shannon diversity (a version of the Shannon diversity index expressed in units of species) using iNEXT 2.0.20 [39]. To obtain comparable estimates of community diversity across lakes, we standardized sampling by estimating Hill–Shannon diversity at 0.95 coverage of the trematode community. Coverage is a measure of the completeness of a sample, as it corresponds to the proportion of individuals in the community that belong to species represented in the sample [40]. Standardizing abundance at equal sample coverage is a superior alternative to the more commonly applied procedures of estimating diversity at equal sample size or asymptotically, because, in contrast with the latter two methods, it also accounts for the distribution of the relative abundances of the species present in the community [40]. The sample size required to reach a certain coverage can vary between communities, meaning that, when comparing community diversity among lakes at equal coverage, the sample sizes for each lake would be unequal. Depending on whether the sampling effort for each community reached the required sample size, our diversity estimates are either based on rarefaction or on extrapolation of the observed data. We also characterized the phylogenetic diversity of each lake’s trematode community by computing the mean pairwise phylogenetic distance (MPD; [41]) among all trematodes recovered from a lake, using picante 1.8.2 [42]. Finally, based on the observed number of parasite infections, we also calculated the adjusted trematode abundance per lake, which accounts for the bias of characterizing parasite infracommunities from *ca* 200 snails in lakes where >200 specimens were collected (§2b). Per lake, we multiplied the observed number of infections by the ratio between the total number of *B. tropicus* collected and those screened for trematode infections.

### (e) Analysis of land use and its effects on trematode communities

All statistical analyses were performed in R 4.3.1 [43] with significance levels fixed at *α* = 0.05 when applicable. First, we used corrplot 0.92 [44] to characterize natural and anthropogenic environmental gradients by computing and visualizing the pairwise Pearson correlation coefficient and associated *p*-value for all pairs of 10 quantitative environmental variables. We also performed principal component analysis (PCA) of all quantitative environmental variables to illustrate patterns of association. Based on the observed correlations between environmental variables (§3a), we tested the effect of land use on the studied host–parasite system using the lakes’ aquatic productivity and the proportion of crop-field cover within crater catchments as anthropogenic variables. We first assessed which of these two variables exerts a dominant influence on the abundance of *B. tropicus* in the 34 studied lakes (electronic supplementary material, equation S1) using generalized additive models (GAMs) with a negative binomial distribution and a logarithmic link function, as is suitable for species count data [45]. All GAMs in this study were fitted by maximizing the marginal likelihood of the model using mgcv 1.9*-*0 [46]. We examined the relationship between *B. tropicus* abundance and our anthropogenic predictors using partial effects (i.e. the relationship between a predictor and the response while keeping the covariates fixed), accounting for associated *p*-values and the proportion of deviance explained by the model (i.e. measure of the goodness-of-fit of GAMs).

Subsequently, we examined how variation in the proportion of crop-field cover and aquatic productivity relate to patterns of parasite abundance and diversity in the 16 lakes where trematode communities were characterized. We fitted GAMs with trematode abundance, species richness estimated at 0.95× coverage, trematode Hill–Shannon diversity at 0.95× coverage and trematode MPD as response variables (electronic supplementary material, equations S2–S5), but now including *B. tropicus* abundance as predictor to test whether it influences parasite communities. For trematode abundance, we fitted GAMs using a negative binomial distribution and a logarithmic link function, whereas for trematode diversity metrics, we used normal distributions.

Finally, we investigated how trematode community composition varies with land use. We focused on the 16 lakes where infections were characterized, but Kyaninga was also excluded from this analysis because none of its 58 *B. tropicus* specimens were infected by trematodes so there is no community to characterize (note that Kyaninga is included in analyses of trematode diversity, having a species richness of zero). For the other 15 lakes, we converted our taxon-per-site abundance matrix to a Bray–Curtis dissimilarity matrix to quantify trematode communities via non-metric multi-dimensional scaling (NMDS) using vegan 2.6*-*4 [47]. Based on the patterns observed in NMDS, we then subjected our Bray–Curtis dissimilarity matrix to distance-based redundancy analysis (db-RDA) to investigate links between trematode community composition and the two predictors representing anthropogenic impact (electronic supplementary material, equation S6). Finally, to understand how anthropogenic impacts propagate through the studied system, we classified the trematode taxa within each community according to their inferred final host(s), namely livestock, birds, multiple final hosts (these are versatile trematode taxa able to infect either mammals, birds or other animals) and unknown host animals. We examined how the anthropogenic variables influence parasite abundance from each group using GAMs with a negative binomial distribution and a logarithmic link function (electronic supplementary material, equations S7–S10).

## 3. Results

### (a) Natural and anthropogenic environmental variation among lakes and their catchments

Measures of environmental variation across our 34 studied lakes (electronic supplementary material, table S1) display multiple patterns of correlation (electronic supplementary material, figure S1). As expected, MAT and MAP are strongly negatively correlated because the crater lakes occupy a geographically restricted area. MAT mainly depends on elevation and decreases with altitude, whereas MAP increases from the rift valley (*ca* 900 m a.s.l.; Bunyaruguru cluster) to the adjacent hillsides (*ca* 1500 m asl; Ndali-Kasenda and Fort Portal clusters). Land use types (timber plantations and crop fields) have weak to modest positive relationships with precipitation because higher rainfall promotes rain-fed agriculture and forestry. Conversely, most lakes protected from anthropogenic disturbance within national parks are located in slightly drier areas at low elevation, because parks have often been established in places that are less suitable for agriculture and thus less populated. Nevertheless, the natural vegetation in these protected lake catchments is mostly forest [28], either moist evergreen tropical lowland forest (Kibale National Park) or dry deciduous tropical lowland forest interspersed with patches of open woodland and tall-grass savannah (Maramagambo Forest in Queen Elisabeth National Park). In this study, such natural grassland is grouped with forest into the category natural vegetation, because its protected status and location within forested areas confer a high soil stability comparable with that in forests. Negative relationships logically link the fractional areas of natural vegetation and those of tree plantations and crop fields within each catchment, as the sum of these land-cover classes equals 100%. Importantly, sediment yield to the lakes reflects soil erosion and is hence a proxy for the excess nutrient input to those lakes resulting from land use within their catchments. It shows significant positive and negative relationships with the fraction of catchment area occupied by, respectively, crop fields and natural vegetation and is also influenced by the crater catchment slope, because steeper terrain facilitates soil erosion after disturbance [28]. Sediment yield is not significantly correlated to the fraction of timber plantations, as soil stability in such plantations is much higher than under any other type of land use in this region [28].

Concerning the aquatic habitat of trematodes and their intermediate snail hosts, surface-water pH displays a strong positive relationship to conductivity and a less strong but still significant positive relationship to aquatic productivity. These correlations indicate that, although only freshwater lakes were included in this study, much of the variation in pH among lakes is caused by differences in lake residence time and hydrological throughflow. Differences in throughflow may also explain the positive versus negative relationships of pH with MAP and MAT, respectively, because higher ambient temperature and lower rainfall tend to increase lake residence time by enhancing the role of evaporation in the annual water balance. The positive relationship between pH and aquatic productivity reflects the significant but lesser influence on pH from CO_2_ uptake by phytoplankton [15]. Aquatic productivity is also positively related to sediment yield, reflecting its dependence on the excess nutrient input caused by soil erosion in the catchment. The observation that various characteristics of the aquatic habitat correlate with the relative proportion of crop fields and natural vegetation in the adjacent catchment highlights the effects of land use on lake functioning as reflected by aquatic productivity. Aquatic productivity and the fraction of catchments occupied by crop fields were therefore used as principal anthropogenic variables in GAM and db-RDA analyses of the effects of land use on *B. tropicus* abundance and on the abundance, diversity and community composition of their trematode parasites.

**Figure 2.**
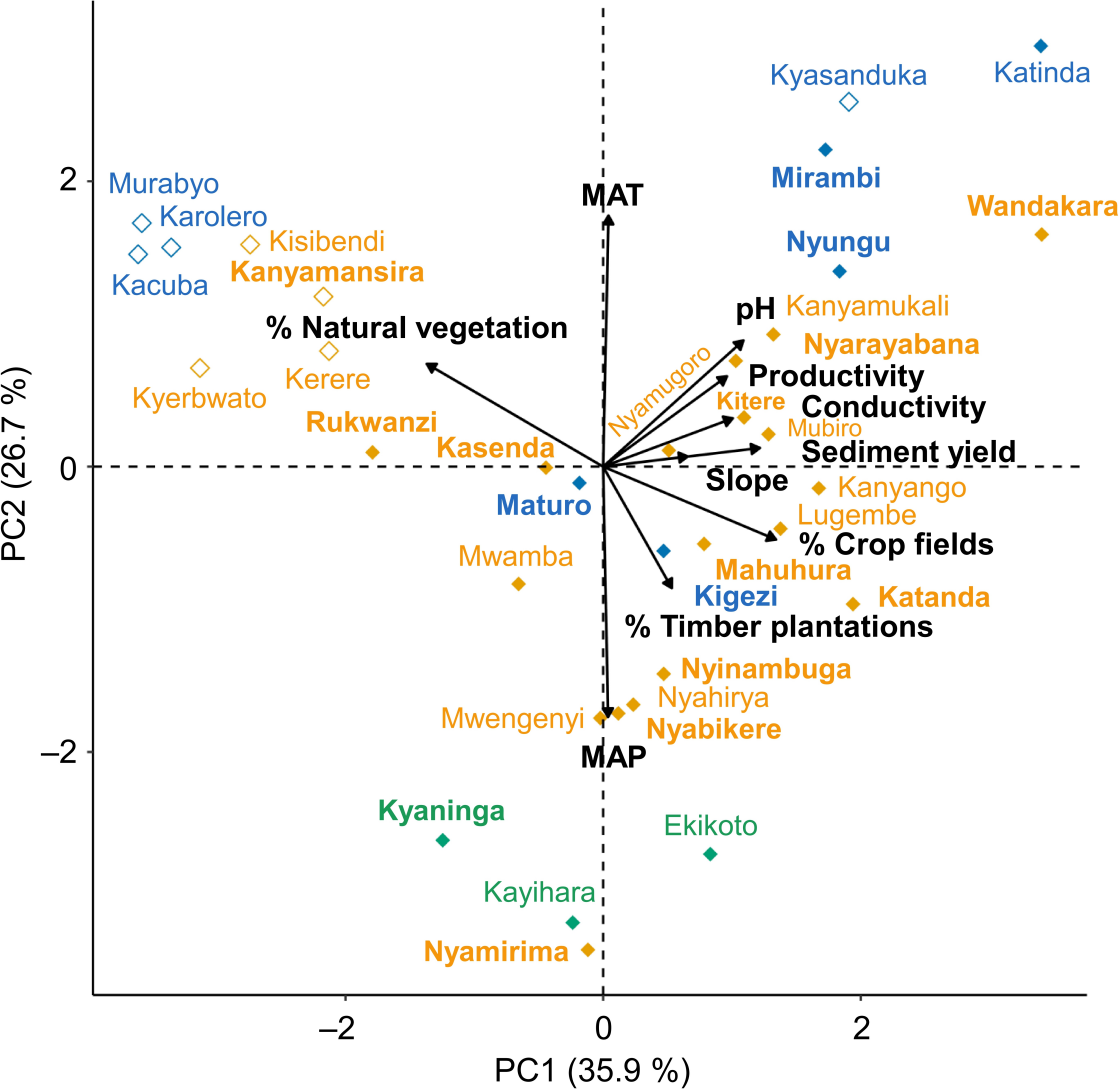
Environmental variation among the 34 studied lakes based on PCA of 10 quantified variables, with PC1 and PC2 together accounting for 63% of the recorded variation. Lakes in green, orange and blue belong to the Fort Portal, Ndali-Kasenda and Bunyaruguru clusters, respectively, with lakes in protected areas indicated by open symbols. The 16 lakes where trematode communities were genetically characterized (i.e. where ≥50 *B. tropicus* specimens were collected) are labelled in bold. MAP, mean annual precipitation; MAT, mean annual temperature.

Our PCA of environmental variation among the 34 studied lakes (figure 2) reveals similar patterns to the correlation analysis (electronic supplementary material, figure S1) but highlights the multivariate nature of observed relationships between variables. PC1 and PC2 together capture 63% of the recorded environmental variation. PC1 predominantly represents variation in anthropogenic disturbance, with unimpacted and heavily impacted lakes having low and high PC1 scores, respectively (figure 2). Unlike all other lakes located in protected areas (open symbols in figure 2), Kyasanduka has a positive PC1 score because its catchment lies largely outside Queen Elisabeth National Park (figure 1) and is there covered by crop fields (electronic supplementary material, table S1). High aquatic productivity and pH are associated with high values for crop-field cover, as mediated by high sediment yield and catchment slopes. PC2 mostly expresses natural environmental variation among the studied lakes, with lowland lakes (mostly of the Bunyaruguru cluster) having higher PC2 scores owing to high MAT and low MAP, whereas lakes at higher altitude (such as all three Fort Portal lakes) have lower PC2 scores.

### (b) Distribution and abundance of the intermediate host *Bulinus tropicus*

**Figure 3.**
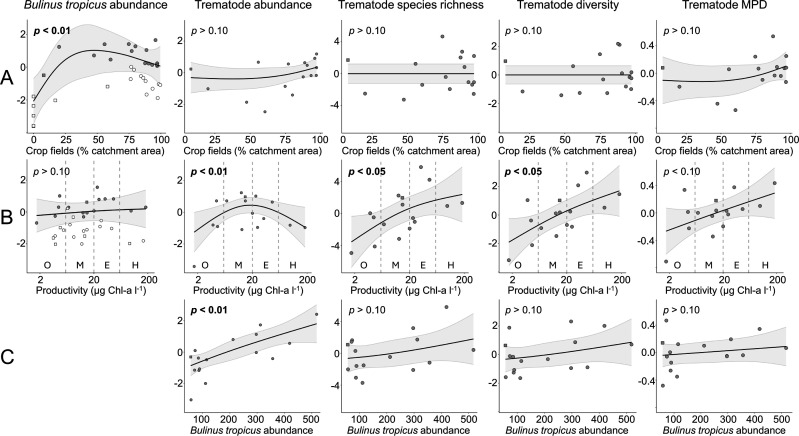
GAM plots displaying the partial (i.e. unique) effects of (A) land use (% catchment area covered by crop fields), (B) lake productivity and (C) *B. tropicus* abundance (for trematode response variables only) on (from left to right) *B. tropicus* abundance, trematode abundance (number of infections in a lake adjusted for effort), trematode species richness estimated at 0.95× coverage, trematode Hill–Shannon diversity estimated at 0.95× coverage and trematode MPD in the 16 Ugandan crater lakes where ≥50 *B*. *tropicus* specimens were collected. Grey shading represents the 95% confidence intervals. The only lake located in a national park is Kanyamansira (square symbol). Lake productivity is represented by phytoplankton biomass, with vertical dashed lines marking the separation between the four classes of trophic status: oligotrophic (O), mesotrophic (M), eutrophic (E) and hypertrophic (H) [15]. Grey symbols indicate the 16 lakes where trematode communities were characterized.

The abundance of *B. tropicus* (i.e. total number of specimens collected per lake) ranges from none in 10 lakes to 518 specimens in Maturo (electronic supplementary material, table S1). Incorporating distribution data from other recent surveys [20,27] documents the presence of *B. tropicus* in 28 of the 34 studied lakes. GAM modelling reveals a negative binomial relationship between *B. tropicus* abundance and the proportion of lake catchment covered by crop fields: abundance increases with land use up to when *ca* 50% of the catchment is covered by crop fields but decreases as land use is intensified further (electronic supplementary material, table S2; figure 3A). Simultaneously, we find a weak and non-significant positive relationship between *B. tropicus* abundance and aquatic productivity (figure 3B).

### (c) Characterization of trematode communities hosted by *Bulinus tropicus*

Of the 16 lakes where *B. tropicus* was sufficiently abundant to genetically characterize the local trematode community (*n* > 50 snails), 11 are located in the Ndali-Kasenda cluster (out of 22 surveyed), four in the Bunyaruguru cluster (out of nine) and one in the Fort Portal cluster (out of three). With exception of Kanyamansira located on the border of Kibale National Park, *B. tropicus* was rare or absent in lakes within protected areas (electronic supplementary material, table S1; figure 1). Out of a total of 2385 *B. tropicus* specimens from these 16 lakes tested for trematode infection, 861 (36.1%) tested positive. These infections occurred in 15 of the 16 lakes analysed, with a prevalence ranging from 0% in Kyaninga (58 snails screened) to 96.2% in Maturo (208 snails screened; electronic supplementary material, table S1). Genetic characterization of the trematode infections by HTAS was successful in 816 of these infected snails (for the remaining 45 specimens, either PCR amplification or Illumina sequencing failed), yielding a total of 1236 fully documented trematode infections. The resulting ASVs belong to 45 different trematode taxa. Of these, 11 could be identified to species based on genetic characterization by other authors (electronic supplementary material, table S3). The 34 remaining taxa were identified to varying supra-specific levels via multi-locus phylogenetic analyses. None of the four mitochondrial markers (cox1 I, cox1 II, cytb and NAD1) suffered from substitutional saturation, but our ITS2 alignment required curation using Gblocks, which resulted in a 196 bp alignment. The partition scheme with the best BIC (Bayesian information criterion) score in ModelFinder [48] consisted of three partitions: one grouping COI1, COI2 and cytb together, whereas NAD1 and ITS2 remained separate. The best-fit substitution models were, respectively, a transition model and a transversion model, both with unequal base frequencies, invariant sites and gamma distribution for rate heterogeneity, and a transversion model with equal base frequencies, invariant sites and gamma distribution for rate heterogeneity. The resulting IQTree multi-locus maximum-likelihood phylogeny (electronic supplementary material, figure S2) allowed us to assign 2, 11 and 21 trematode species to genus, family and superfamily/suborder level, respectively. Reference to published records indicated that among the 11 trematodes identified to species level, three have cattle as final host, seven infect fishes as second intermediate host and birds as the final host and one infects amphibians (electronic supplementary material, table S3). Among the other 34 trematode species, 9 belong to groups that typically infect fishes as second intermediate host and either birds or mammals as final hosts; three others belong to genera that infect a broad range of final hosts (including birds, mammals and amphibians), whereas for the remaining 22 taxa no specific final host(s) could be established owing to insufficient taxonomic resolution. The abundance of parasite infections (adjusted for sampling differences) in each of these 16 lakes ranged from 0 (Kyaninga) to 1078 (Maturo; electronic supplementary material, table S3) and parasite species richness from 0 to 16. Five trematode species (*Petasiger* sp. 5 ML, *Patagifer* sp. 1 ML, *Echinoparyphium* sp. ML, *Euclinostomum heterostomum* and Plagiorchioidea sp. 1) are both widespread (each occurring in between five and 11 of the studied lakes) and abundant (representing together 49.9% of all infections). Three of these taxa (*Petasiger* sp. 5, *Patagifer* sp. 1 and *E. heterostomum*) infect birds, and *Echinoparyphium* sp. likely infects both birds and mammals; the final host(s) of Plagiorchioidea sp. 1 is/are unknown. Contrastingly, most other trematode species were found at low abundance and often in only one (27 taxa) or a few lakes. The composition of parasite communities differed greatly among lakes: Bray–Curtis pairwise dissimilarities ranged from 0.29 to 1.00 in our dataset, averaging 0.88 ± 0.14 (mean ± s.d.). Finally, in four *B. tropicus* specimens from Nyarayabana and Katanda, we identified *Fasciola gigantica*, a cattle parasite that more typically infects snails of the family Lymnaeidae (which in the Ugandan crater lakes is represented by *Radix*) as intermediate host.

### (d) Determinants of trematode abundance and community diversity and composition

GAMs indicate that the abundance of parasitic trematodes in lakes has a significant, hump-shaped relationship with aquatic productivity, with maximal abundances around the transition from mesotrophic to eutrophic lakes (approx. 20 µg l^–1^ Chl-a; figure 3A and electronic supplementary material, table S2). Trematode abundance increases more linearly with *B. tropicus* abundance (figure 3B) and also has a positive but non-significant relationship with the fraction of crop fields in lake catchments (figure 3C). The species richness and Hill–Shannon diversity of trematode communities in the lakes with infected *B. tropicus* (i.e. excluding Kyaninga) could be predicted by rarefaction for all lakes except Nyungu, Wandakara, Nyabikere and Kitere, where modest extrapolation was required (electronic supplementary material, figure S3). Estimated trematode species richness varies between 0 and 12.5, and Hill–Shannon diversity varies between 0 and 6.7, whereas trematode phylogenetic diversity (MPD) ranges from 0 to 1.4 (electronic supplementary material, table S3). GAM modelling shows that trematode species richness and Hill–Shannon diversity increase significantly with aquatic productivity (electronic supplementary material, table S2), albeit that the increase in raw species richness levels off in the most productive lakes (>20 µg l^−1^ Chl-a; figure 3B). Trematode phylogenetic diversity is also positively related to aquatic productivity (figure 3B), but this relationship is not statistically significant (electronic supplementary material, table S2). All three measures of trematode diversity also show a positive (but not statistically significant) relationship with the local abundance of *B. tropicus* (electronic supplementary material, table S2; figure 3C). In contrast, no relationship is found between any of these three trematode diversity measures and the areal fraction of catchments covered by crop fields (electronic supplementary material, table S2; figure 3A).

**Figure 4.**
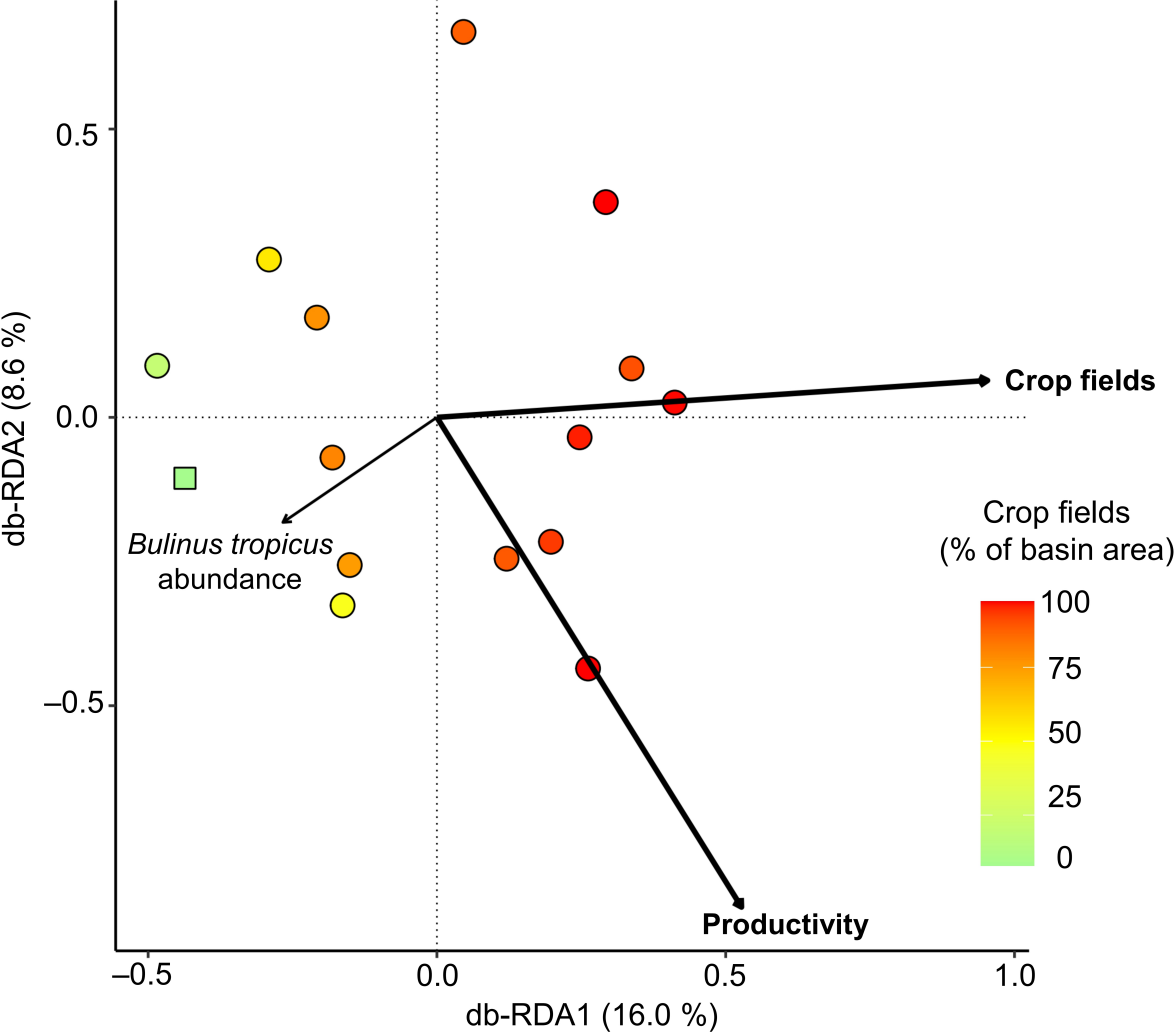
Distance-based redundancy analysis (db-RDA) applied to the Bray–Curtis dissimilarity matrix of the trematode communities infecting *B. tropicus* in 15 Ugandan crater lakes, showing the impact of land use, as expressed via aquatic productivity and the fraction of crop fields in catchments, as well as host snail abundance on trematode community composition. Lakes of the Ndali-Kasenda cluster are represented by circles, and those of the Bunyaruguru cluster by squares. The two variables with significant effect on community dissimilarity are highlighted in bold.

The results of the NMDS (electronic supplementary material, figure S4) and db-RDA (figure 4) both show that the substantial variation in trematode community composition among the studied lakes relates to differences in anthropogenic land use in lake catchments and in aquatic productivity. The db-RDA of the Bray–Curtis community dissimilarity matrix onto the anthropogenic environmental variables and *B. tropicus* abundance yields a significant *p*-value for the overall model (<0.01) and explains 30.7% of all compositional variance in the studied trematode communities. Both the percentage of crop fields in catchments and lake productivity have a significant influence on trematode community composition, whereas the local abundance of *B. tropicus* in these lakes does not (electronic supplementary material, table S2). The db-RDA results (figure 4) indicate that the 15 trematode communities are arranged along the first ordination axis according to crop-field cover, i.e. the relative intensity of land use, whereas compositional variation related to aquatic productivity, i.e. how land use affects nutrient recycling in lakes, is projected on both db-RDA1 and db-RDA2. Lakes in more disturbed catchments display larger variability in trematode community composition along db-RDA2 than those in more natural catchments (figure 4).

**Figure 5.**
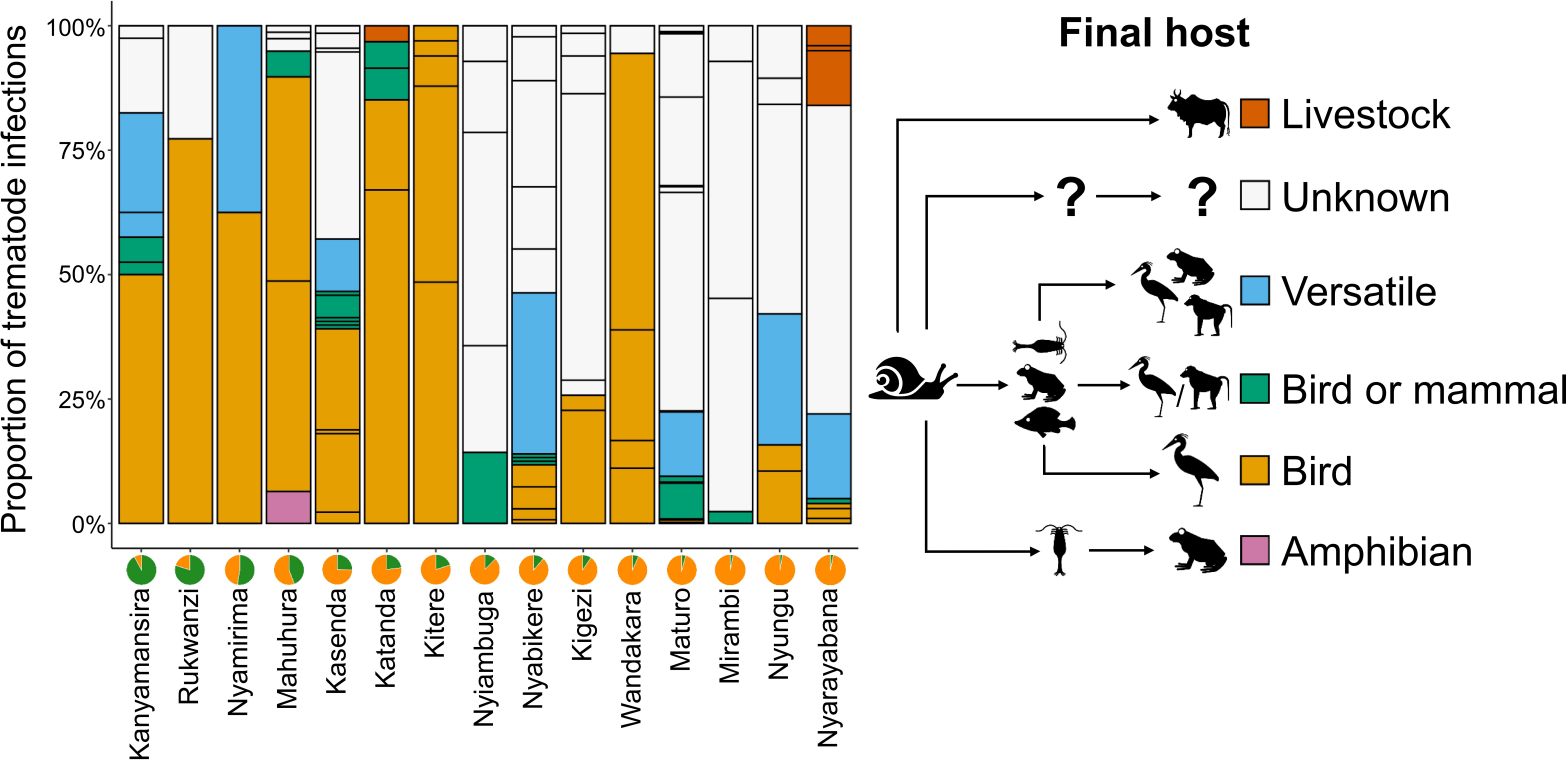
Proportion of trematode communities in each of 15 studied lakes associated with five major categories of known or inferred final host. Lakes are organized from left to right by increasing fraction of their catchment covered by crop fields, as shown in the pie charts (green = natural vegetation + timber plantations; orange = crop fields). The figure legend is augmented by a simplified schematic of the respective trematode life cycles (see electronic supplementary material, table S3 for details).

How trematode community composition varies with land use is illustrated in figure 5 and evidenced by variable GAM responses of the abundance of the four subsets of trematodes with different final hosts (electronic supplementary material, tables S2 and figure S5). The proportion of trematodes with unknown final hosts (53.2% of all infections) increases significantly with the fraction of crop-field cover in the catchment and has a significant hump-shaped relationship with aquatic productivity. The abundance of trematodes infecting diverse final hosts (versatile, 12.1% of all infections) does not vary with land use, but increases significantly with aquatic productivity before levelling off in hypertrophic lakes. Contrastingly, trematodes infecting birds (26.9% of all infections) decrease significantly in catchments that are largely (>75%) covered by crop fields, but their abundance does not vary with aquatic productivity. Finally, the abundance of trematodes infecting livestock (1.5% of infections) does not vary significantly with either productivity or land use, which could be owing to limited statistical power as the two lakes where such parasites were found (Katanda and Nyarayabana) are both mesotrophic and have most of their catchment covered by crop fields.

## 4. Discussion

### (a) The Ugandan crater lakes as natural laboratory for study of trematode ecology

Our environmental characterization of the 34 studied lakes and their catchments confirms that they only display modest natural environmental variation [14,15], mainly owing to their geographic proximity and uniform geology. Agricultural conversion since the 1950s developed heterogeneously across the region, such that the catchments of most lakes are now settled to varying degrees by communities practising mixed subsistence farming and cattle herding, while some became protected in national parks and forest reserves. Consequently, these lakes provide a unique natural laboratory to study the influence of anthropogenic activities on aquatic and terrestrial ecosystems, with limited interference from confounding factors [14,25]. In this study, lakes were selected to maximally cover the regional variation in anthropogenic impact both on land and in the water. Given the general lack of historical monitoring data that would allow a longitudinal analysis of anthropogenic impact, this cluster of quasi-replicated tropical ecosystems provides invaluable data on how anthropogenic stressors affect parasitic trematodes.

### (b) Agricultural land use promotes the intermediate host snails, up to a point

We found a significant non-linear relationship between *B. tropicus* abundance and the fraction of crop fields in lake catchments: abundance increases with land use until *ca* 50% of the catchment is covered by crop fields but declines at higher land use intensity (figure 3). We also found a positive, non-significant association of *B. tropicus* abundance with aquatic productivity (figure 3; electronic supplementary material, table S2). These results confirm findings from earlier studies [22,23] showing that moderate anthropogenic disturbance favours eurytopic snails such as *B. tropicus*, likely because they benefit from the increased abundance of suitable littoral habitat and food (epiphytic algae and fine organic detritus), which results from the enhanced nutrient input associated with agriculture. Additionally, pulmonate snails of Bulinidae and Planorbidae have short generation times and can tolerate hypoxia associated with organic pollution [21], which are key advantages that explain, at least partly, their success in organically polluted freshwater habitats. Consequently, agricultural land use may convert lakes into trematode transmission hotspots [23], at least under moderate levels of organic pollution. High land use intensity causes excessive sediment and nutrient input to freshwater lakes and tends to eradicate submerged aquatic macrophytes, a critical state transition [49] often resulting in hypertrophic conditions with dense blooms of noxious phytoplankton, bottom anoxia and a general degradation of benthic habitats including those inhabited by *Bulinus* species.

### (c) Aquatic productivity and host snail abundance shape trematode abundance and diversity

Our finding that the abundance of snail-borne trematodes in Ugandan crater lakes increases with the local abundance of their intermediate host *B. tropicus* is consistent with earlier studies linking parasite and host abundance in snail-trematode systems [50], albeit that such links can be complex. On the one hand, high snail density can increase the probability that parasite larvae find a suitable snail host, thereby enhancing the likelihood of successful transmission and increasing parasite abundance. However, experimental evidence suggests that high snail density decreases the infection risk of individual snails when the increase in infection rate is lower than that of snail density [50]. On the other hand, high trematode prevalence in snail populations may negatively affect snail density owing to reduced snail reproductive success [51], which could subsequently reduce snail population size, limiting the trematode infection rate and eventually reduce local trematode abundance. Our data do not allow us to pinpoint the exact mechanism(s) responsible for the positive relationship between snail and parasite abundance, but they confirm the overall positive influence of host density on parasite transmission dynamics. As *B. tropicus* is most abundant in lakes with catchments under intermediate crop-field cover, we might also expect the highest parasite abundances in these lakes. However, we found no significant correlation between parasite abundance (or diversity) and crop-field cover, but instead a strong, significant relationship with aquatic productivity. For trematode abundance that relationship is hump-shaped, whereas it is positive throughout the productivity gradient for all three diversity measures. The initial increase of trematode abundance with increasing productivity suggests that intermediate and final hosts may be more abundant in mesotrophic and slightly eutrophic lakes, therewith increasing transmission dynamics [47]. The drop in parasite abundance in the most productive lakes possibly reflects a high sensitivity of free-living trematode larvae to hypoxia, which may result in reduced survival, lower local abundance and hence lower transmission [47,52]. To our knowledge, our study is the first to document a significant positive correlation between trematode diversity in intermediate host populations and aquatic productivity. Such a diversity–productivity relationship had been postulated as logical consequence of the higher abundances and diversity of intermediate and final hosts in more productive systems [53], but to date empirical support has been scarce. For example, no significant correlation was found between the diversity of helminth and crustacean fish parasites and marine productivity in the Line Island archipelago [54]. The few other host–parasite systems where a positive correlation has been found are between the diversity of parasitic arthropods in grasslands and plant productivity [55] and between the diversity of haemosporidian parasites and bird host diversity, which itself is correlated with primary plant productivity [56]. Our results indicate that, at least in a rural tropical setting where trematode-mediated diseases represent a significant health burden, the eutrophication of lakes explains a sizable portion of the variation in trematode abundance and diversity, whereas the abundance of their intermediate host is better explained by the degree of catchment disturbance. Despite a slight decrease in *B. tropicus* abundance as the proportion of crop fields in the catchment increases above 50%, and despite a decrease in trematode abundance as heavily impacted lakes transition to a hypertrophic state, the diversity of trematodes being transmitted continues to increase. This implies that intensive land use increases the overall disease burden for wildlife, livestock and humans, and it underscores the challenges associated with increased risk and decreased predictability of disease outbreaks.

### (d) Trematode communities are shaped by both terrestrial and aquatic stressors

Among the 45 different trematode taxa recovered in 1236 detected infections, only 11 could be identified to the species level; for the other 34, no closely matching genetic sequences are available in current databases. This well-known ‘barcoding void’ originates from the general lack of genetic data on the large majority of named parasite species and a plethora of still undescribed species [57]. Moreover, only a handful of the 45 recorded trematode species were both widespread and common, whereas many species were only represented by a few infections in a single lake. High Bray–Curtis dissimilarity of trematode communities between most pairs of lakes similarly indicates that few species are shared among multiple lakes. Such a distributional pattern has been observed in other snail-borne parasite communities and may relate to differences in the abundance and aggregation of intermediate and final hosts [58].

The results of our db-RDA (figure 4) show that much variation in trematode community composition among lakes is explained by variation in crop-field cover within their catchments. This finding is consistent with those of [10] and [59] on links between land use and parasite communities of bats and frogs, respectively. In our case, variation in trematode community composition also correlates with aquatic productivity of the lakes adjacent to those crop fields. Thus, agricultural land use shapes the community composition of parasitic trematodes both directly (impact on land) and indirectly (eutrophication of the aquatic habitat of their intermediate host). The direct effects must relate to the suitability of riparian habitat for final hosts with a predominantly terrestrial lifestyle [9]. Lack of information on the ecology of individual trematode taxa at various stages in their complex life cycle and on the identity of their final hosts (and in some cases also secondary intermediate hosts) hampers discrimination between the two types of effects. Nevertheless, our results reveal contrasting relationships among trematode subgroups associated with different categories of final host and land use intensity. Trematodes infecting birds are most abundant in catchments with intermediate crop-field cover, perhaps because such catchments offer the highest combined diversity of natural and anthropogenic habitats [60] and may thus offer birds a greater variety of habitats, food or prey [53]. The striking collapse in the proportion of bird parasites in lake catchments with >80% crop-field cover (figure 5) may then reflect a wholesale deterioration of local feeding and nesting areas. Contrastingly, no correlation between trematode abundance and crop-field cover was found for ‘versatile’ (i.e. multi-host) parasites, possibly because these trematodes can complete their life cycle in hosts occurring in either natural or anthropogenic environments [61].

The high proportion of trematodes that infect large domestic livestock in Nyarayabana (figure 5: 16% of the total trematode burden) coincides with this lake being one of four with a catchment almost fully converted to crop fields (>95%; electronic supplementary material, table S1; figure 5), as well as being one of three lakes (together with Kitere and Mahuhuru) that are regularly visited by transhumant pastoralists to water large herds of cattle [62]. Livestock density is evidently not the sole factor determining the prevalence of livestock-infecting trematodes in local *B. tropicus* populations, considering the absence of such parasites in Kitere and Mahuhura (although at least three such species occur in the region; electronic supplementary material, table S3). Katanda, the only other lake where we found a known livestock-infecting trematode (*F. gigantica*), has a crop-field cover of 77.1% (electronic supplementary material, table S1) but a relatively modest number of resident livestock [62]. Lastly, parasites with multiple intermediate host organisms can be expected to be most impacted by intensive land use because each host may be vulnerable to a different aspect of habitat deterioration [10,63]. Fragmentary knowledge of parasite life cycles does not allow us to test this hypothesis in this study, but in line with the general simplification of food webs and ecosystem functioning in heavily disturbed ecosystems, we expect trematodes with simple life cycles to be overrepresented in the most heavily impacted lakes [64].

### (e) Land use impacts parasite transmission dynamics and ecosystem health

The results of this study indicate that the intensification of agriculture in rural western Uganda increases the risk of trematode parasite transmission to wildlife, fisheries, domestic livestock and humans, as has been found elsewhere [5]. In addition, our results demonstrate that the impact of land use differs among groups of trematodes with different final hosts. Species that can infect a broad spectrum of final hosts become more abundant with increasing agricultural intensity. Contrastingly, those infecting host whose local habitat is negatively impacted by agricultural land use (birds or other wild animals) decline. The regional presence of *Calicophoron* sp., *Schistosoma bovis* and *F. gigantica,* which we document, clearly demonstrates that trematode transmission to livestock is also already ongoing, and this will likely increase as agricultural land use intensifies, considering that the two lakes where we observed livestock infections are largely surrounded by crop fields. Additionally, given that many trematode species infect fishes either as final or intermediate host (figure 5), increased parasite transmission in eutrophied lakes may also negatively impact the cage aquaculture, which is now being pursued in some lakes as part of a government strategy to boost their economic value [63].

Finally, besides *B. tropicus*, the Ugandan crater lakes harbour several other eurytopic snail species, including *Biomphalaria sudanica* and *Biomphalaria pfeifferi*, which are responsible for transmitting *Schistosoma mansoni* [20,27], the causative agent of human intestinal schistosomiasis. If our results on the effects of land use on the trematode communities associated with *B. tropicus* also apply to these other snail species, which is highly likely considering an analysis of aquatic snail communities in Lake Victoria [22], then further intensification of agricultural activity in western Uganda may create opportunities for transmission of human schistosomiasis [23]. The links between land use and trematode transmission documented here should constitute a warning that land use choices made to meet humanity’s growing resource demands are destined to shape trematode communities and, therefore, will define tomorrow’s hotspots of parasite transmission as well as which hosts will be most affected. We therefore encourage local stakeholders, research institutes and authorities to upscale parasite monitoring initiatives according to the One Health principle, thus not only focusing on human and livestock parasites but also on those of fish and wildlife.

## Data Availability

Environmental data, trematode ASV matrix and details on trematode species: [65]. R script for the analysis of trematode diversity: [66]. Quality-controlled DNA sequences of trematode parasites: GenBank accession numbers OQ548105-OQ549888 (ITS2); OQ543469-OQ543563 (cox1 I); OQ606413-OQ606758 (cox1 II); OQ573734-OQ574607 (NAD1) and OQ574626-OQ575328 (cytb). Raw Illumina sequencing read sequences: Sequence Read Archive accession PRJNA1227448. Electronic supplementary material is available online [67].
